# Do early‐life exposures explain why more advantaged children get eczema? Findings from the U.K. Millennium Cohort Study†

**DOI:** 10.1111/bjd.14310

**Published:** 2016-02-23

**Authors:** D.C. Taylor‐Robinson, H. Williams, A. Pearce, C. Law, S. Hope

**Affiliations:** ^1^ Department of Public Health and PolicyWhelan BuildingUniversity of LiverpoolLiverpoolL69 3GBU.K.; ^2^ UCL Institute of Child Health30 Guilford StreetLondonWC1N 1EHU.K.; ^3^ Centre of Evidence Based DermatologyUniversity of NottinghamNottinghamU.K.

## Abstract

**Background:**

Atopic dermatitis (eczema) in childhood is socially patterned, with higher incidence in more advantaged populations. However, it is unclear what factors explain the social differences.

**Objectives:**

To identify early‐life risk factors for eczema, and to explore how early‐life risk factors explain any differences in eczema.

**Methods:**

We estimated odds ratios (ORs) for ever having had eczema by age 5 years in 14 499 children from the U.K. Millennium Cohort Study (MCS), with a focus on maternal, antenatal and early‐life risk factors and socioeconomic circumstances (SECs). Risk factors were explored to assess whether they attenuated associations between SECs and eczema.

**Results:**

Overall 35·1% of children had ever had eczema by age 5 years. Children of mothers with degree‐level qualifications vs. no educational qualifications were more likely to have eczema (OR 1·52, 95% confidence interval 1·31–1·76), and there was a gradient across the socioeconomic spectrum. Maternal atopy, breastfeeding (1–6 weeks and ≥ 6 months), introduction of solids under 4 months or cow's milk under 9 months, antibiotic exposure in the first year of life and grime exposure were associated with an increased odds of having eczema. Female sex, Pakistani and Bangladeshi ethnicity, smoking during pregnancy, exposure to environmental tobacco smoke and having more siblings were associated with reduced odds for eczema. Controlling for maternal, antenatal and early‐life characteristics (particularly maternal smoking during pregnancy, breastfeeding and number of siblings) reduced the OR for eczema to 1·26 (95% confidence interval 1·03–1·50) in the group with the highest educational qualifications compared with the least.

**Conclusions:**

In a representative U.K. child cohort, eczema was more common in more advantaged children. This was explained partially by early‐life factors including not smoking during pregnancy, breastfeeding and having fewer siblings.

## Abstract 2

**What's already known about this topic?**

- Previous studies have suggested that atopic dermatitis (eczema) in childhood is socially patterned, with higher incidence in more advantaged populations.
- It is unclear what explains these differences.

**What does this study add?**

- This large longitudinal study confirmed a significant excess of atopic dermatitis in more advantaged children born in 2000.
- This could be explained partially by maternal and early‐life factors including less maternal smoking in pregnancy, prolonged breastfeeding and fewer siblings.

Plain language summary available online

## Introduction

Childhood eczema is the most common inflammatory skin disease, and previous estimates suggest that it affects around 20% of children in the U.K.^1^ The condition is also referred to as atopic dermatitis and atopic eczema. It is characterized by itch, skin inflammation, a skin barrier abnormality and susceptibility to skin infection.^2^ Childhood eczema can have a serious effect on children's and families’ quality of life, for example through a negative impact on sleep, and has been shown to influence schooling.^2^ The resulting impairment in health‐related quality of life is comparable with that of other chronic diseases of childhood, including diabetes and asthma.^3^

The aetiology of eczema is poorly understood. There is clearly a genetic component involving skin barrier proteins such as filaggrin,^4^ but previous studies have highlighted the need to investigate further the social and environmental factors associated with the development of childhood eczema.^5^ Given the large public health burden and unclear risk factor profile, it is important that studies identify potentially modifiable risk factors for eczema that are amenable to public health intervention, and there is increasing evidence that early‐life environmental exposures may be particularly important.^6^

One interesting feature of previous epidemiological studies of eczema is the finding of a reverse social gradient, with higher prevalence in children from more advantaged circumstances.^7^, ^8^ The reasons for the reverse social gradient are unclear. Higher prevalence rates of allergies in more privileged social groups have been considered consistent with the hygiene hypothesis,^9^ and understanding this phenomenon may provide broader insights into the aetiology of eczema.

Our objectives were to identify early‐life risk factors for eczema and to explore how early‐life risk factors explain any differences in eczema by socioeconomic circumstances (SECs). We hypothesized that a range of previously identified early‐life exposures may be risk factors for eczema; that the reverse social gradient found in older cohort studies still exists in modern U.K. society;^7^, ^8^ and that this might be attenuated by early risk factors for eczema, including weaning patterns and early exposure to infections that may differ by SEC.

## Patients and methods

### Design, setting and data source

We used data from the Millennium Cohort Study (MCS), a nationally representative sample of children born in the U.K. between September 2000 and January 2002. Data were downloaded from the U.K. Data Archive in 2014. The study oversampled children living in disadvantaged areas and those with high proportions of ethnic minority groups by means of a stratified clustered sampling design.^10^ Trained interviewers carried out home‐based survey interviews with the main respondent (usually the mother) and their partners (if present). Further information on the cohort and sampling design can be found in the cohort profile.^10^ This study uses data collected on children at ages 9 months and 5 years. The analysis did not require additional ethical approval.

### Outcome measure

At 5 years, when most children at risk of eczema will have developed symptoms,^11^ mothers were asked about whether their child had ‘ever had eczema’. This question was taken from the International Study of Asthma and Allergies in Childhood (ISAAC) core questionnaire.^12^ This validated instrument has been widely used to measure childhood atopic conditions. At age 3 years the question is ‘ever had eczema/hay fever’, and therefore the primary outcome was measured at 5 years.

### Early‐life risk factors

The primary exposure of interest was the highest maternal qualification achieved (National Vocational Qualifications or equivalent groups), used as a fixed measure of SECs at birth. Other prespecified risk factors in the MCS potentially associated with eczema, identified on the basis of a literature review,^6^, ^13^, ^14^, ^15^ included the following. (i) Sex of the child and ethnicity; (ii) Maternal characteristics fixed at birth: age at birth of the MCS child; maternal history of atopy (none, asthma or eczema, asthma and eczema) and maternal prepregnancy body mass index (underweight, normal, overweight, obese, morbidly obese); (iii) Perinatal factors and exposures during pregnancy: maternal smoking (never, smoked before pregnancy, smoked before and during pregnancy – to capture timing – and also amount smoked during pregnancy to assess dose–response effects: never, 1–9 cigarettes per day, 10–19 per day, ≥ 20 per day); caesarean section (yes/no); preterm birth (< 37 weeks’ gestation; yes/no); low birthweight (< 2·5 kg; yes/no); breastfeeding (never, ≤ 1 week, 1–6 weeks, 6 weeks to 6 months, > 6 months); exposure to cow's milk by 9 months (yes/no); early introduction of solid food (coded as < 4 months; yes/no as per Department of Health guidance at the time of the survey); and use of antibiotics (yes/no); (iv) Early‐life postnatal exposures measured at 9 months, including parental smoking in the same room (environmental tobacco smoke, ETS; yes/no); damp or condensation in the home (yes/no); exposure to pollution or ‘grime’ in the residential area (yes/no, based on maternal report); furry pets in the household (yes/no)^16^ and the number of siblings in the household and childcare use (informal and formal) to capture potential exposure to infections.

### Analysis strategy

We first explored the association between eczema and all risk factors, and between maternal education (as the primary exposure of interest) and the other risk factors. Following this we estimated the unadjusted odds of eczema at age 5 years on the basis of the covariates of interest using logistic regression. Then sequential models were fitted, calculating adjusted odds ratios (ORs) for eczema on the basis of maternal education (with children of mothers with no educational qualifications as the reference group), mutually adjusting for the other early‐life risk factors that were significantly associated with eczema at the *P* < 0·1 level in the univariate analysis. We used a life‐course approach to construct the adjusted models, first adding maternal characteristics fixed before birth (considered confounders), then perinatal factors and exposures during pregnancy, and finally postnatal exposures, to show the impact on the association between SECs and eczema. Any observed change in ORs on the addition of risk factors in a final complete case sample, after adjustment for confounders, was taken to indicate potential mediation.^17^ We visualized the change in ORs comparing mothers with the highest qualifications with those with the lowest (the SEC gap).^18^ Analyses were conducted in Stata/SE v.13 (Stata Corporation, College Station, TX, U.S.A.) with svy commands to account for the sample design and attrition. We estimated all model parameters by maximum likelihood.

We undertook a range of prespecified sensitivity analyses to explore the key findings, repeating the analysis with alternative measures of SECs, dropping covariates with larger amounts of missing data. In response to our initial findings we undertook additional analyses exploring the relationship between maternal smoking during pregnancy, breastfeeding and eczema. Because ORs can overestimate associations when the exposure is common,^19^ we also repeated the analysis using risk ratios to check that the conclusions were unchanged. We also fitted the most parsimonious final model, dropping nonsignificant variables in a backwards stepwise manner. To explore interactions between SECs and other covariates in the final model we fitted separate models including each one‐way interaction between maternal education and the other variables in the model.

## Results

In total 14 499 children were present at both 9 months and 5 years and had data on eczema status, and 11 537 (80%) had full data on all exposures of interest in the fully adjusted model. Overall 35·1% of children had ever had eczema by age 5 years. The period prevalence of eczema was higher in children whose parents had higher educational qualifications. All other covariates of interest, except for sex, maternal atopy and introduction of cow's milk by 9 months, varied by level of maternal education (Table 1).

**Table 1:** Characteristics of the total study population, by level of maternal education at the birth of the child (*n* = 14 499)

	Degree plus (*n* = 2542), 18·0%	Diploma (*n* = 1330), 9·4%	A levels (*n* = 1437), 9·7%	GCSE A–C (*n* = 4990), 35·7%	GCSE D–G (*n* = 503) 11·0%	None (*n* = 1552), 6·1%	Total (*n* = 14 499)	*P*‐value^a^
Eczema at age 5 years	39·5	39·0	35·4	34·5	33·6	30·0	35·1	< 0·001
Female	49·7	47·9	49·8	48·4	48·9	49·0	48·9	0·89
Ethnicity								
White	87·3	89·9	89·5	91·9	91·5	74·4	87·8	< 0·001
Mixed	4·2	2·7	2·4	2·7	2·6	4·4	3·2	
Indian	2·5	1·8	2·0	0·9	1·1	2·6	1·7	
Pakistani and Bangladeshi	1·5	1·3	3·1	2·3	2·9	11·4	3·7	
Black or black British	2·9	3·6	1·9	1·5	1·8	4·9	2·6	
Other	1·6	0·7	1·1	0·5	0·1	2·3	1·0	
Maternal age at MCS child's birth (years)								
14–19	0	0·9	4·2	8·1	16·1	15·9	7·7	< 0·001
20–24	2·7	10·0	15·6	19·1	26·7	24·7	16·6	
25–29	24·8	32·5	30·2	28·5	31·1	25·6	28·2	
30–34	43·3	37·2	32·3	29·5	18·4	21·0	30·4	
35–39	25·7	17·4	14·8	13·1	6·9	11·0	15·0	
≥ 40	3·5	2·0	2·9	1·7	0·8	1·8	2·1	
Maternal atopy								
Asthma or eczema	21·3	25·5	22·0	24·0	24·3	22·3	23·2	0·11
Asthma and eczema	5·7	5·1	6·9	6·5	6·8	6·0	6·2	
Maternal body mass index								
Underweight	3·0	3·0	3·5	5·2	8·4	9·6	5·3	< 0·001
Normal range	75·6	68·6	67·3	63·8	60·9	59·7	66·0	
Overweight	15·3	19·9	20·2	21·1	20·0	21·8	19·8	
Obese	5·2	5·9	6·0	7·3	7·2	6·2	6·4	
Morbidly obese	0·8	2·6	3·0	2·6	3·5	2·8	2·4	
Smoking								
Never	87·4	76·1	75·3	60·2	48·9	46·1	64·7	< 0·001
Smoked before pregnancy	9·4	14·8	14·6	18·2	17·3	12·4	14·9	
Smoked during pregnancy	3·2	9·1	10·1	21·7	33·8	41·5	20·4	
Alcohol during pregnancy	48·8	38·8	35·0	30·9	26·6	21·9	33·7	< 0·001
Low birthweight	3·6	5·6	5·5	6·0	6·3	10·2	6·1	< 0·001
Caesarean section	24·9	24·1	19·6	20·0	19·0	17·0	20·6	< 0·001
Breastfeeding								
Never	6·9	17·9	18·4	34·9	46·5	51·7	30·5	< 0·001
≤ 1 week	7·9	13·0	11·1	13·2	16·9	13·6	12·5	
1–6 weeks	9·9	14·1	15·0	15·3	13·4	10·8	13·3	
6 weeks to 6 months	19·7	20·2	20·7	16·8	11·4	10·7	16·5	
> 6 months	55·6	34·7	34·7	19·8	11·8	13·3	27·3	
Cow's milk exposure < 9 months	53·8	54·7	54·0	55·2	56·7	53·0	54·6	0·48
Introduced solids before 4 months	27·4	36·0	37·3	37·6	40·7	36·0	35·7	< 0·001
Antibiotics at age < 1 year	39·9	40·8	39·8	39·7	40·1	37·3	39·5	< 0·001
ETS exposure	2·9	4·7	7·6	12·8	22·5	27·1	13·0	< 0·001
Exposure to ‘grime’	21·2	19·3	19·2	20·4	20·6	26·7	21·3	0·002
Furry pets in household	29·1	37·8	33·2	39·8	39·6	33·3	35·9	< 0·001
Number of children in household								
1	49·7	45·3	45·3	40·8	44·4	30·8	42·1	< 0·001
2–3	47·0	51·2	51·2	53·0	49·1	52·3	51·0	
≥ 4	3·3	3·5	3·5	6·3	6·4	16·9	6·9	
Childcare type at 9 months								
None	49·7	59·9	61·0	69·8	77·6	86·9	67·9	< 0·001
Informal	14·8	21·7	21·7	21·7	17·6	12·0	18·5	
Formal	35·5	18·4	17·2	8·5	4·8	1·2	13·6	

GCSE, General Certificate of Secondary Education; MCS, Millennium Cohort Study; ETS, environmental tobacco smoke. All figures are percentages adjusted for sampling design. Missing data (unadjusted frequency) are ethnicity 3, maternal age 527, maternal atopy 536, maternal body mass index 2622, maternal smoking 561, alcohol during pregnancy 1898, low birthweight 1908, caesarean section 511, breastfeeding 511, cow's milk 514, early solids < 4 months 514, antibiotics 1420, ETS exposure 512, grime exposure 630, furry pet exposure 1413, siblings 511, childcare type 562. ^a^χ^2^‐test.

### Association of covariates with eczema

**Figure 1:**
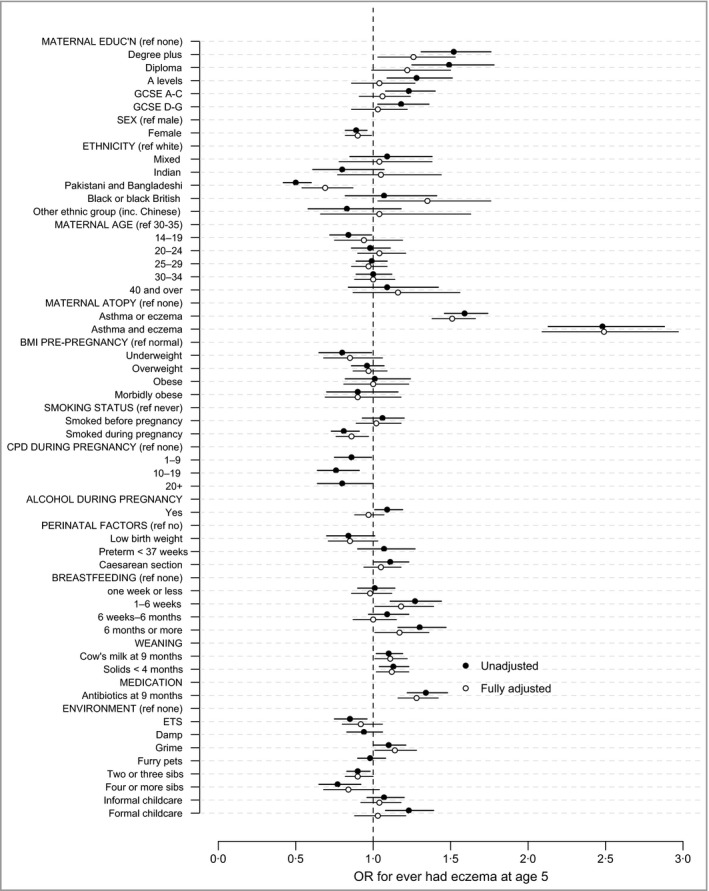
Univariate and fully adjusted associations (odds ratios, ORs) between covariates and eczema. GCSE, General Certificate of Secondary Education; BMI, body mass index; CPD, cigarettes per day; ETS, environmental tobacco smoke.

In univariate regression, higher maternal qualifications, maternal atopy, breastfeeding (1–6 weeks and ≥ 6 months), cow's milk exposure at < 9 months, introduction of solids at < 4 months, antibiotics in the first year of life, exposure to grime and formal childcare were all associated with an increased OR for eczema (Table 2 and Fig. 1). Female sex, Pakistani and Bangladeshi ethnic origin, teenage parenthood, maternal underweight, smoking during pregnancy, ETS exposure and other siblings were associated with a lower OR for eczema.

**Table 2:** Prevalence of ever having had eczema by age 5 years and univariate odds ratios (ORs)

	Total (*n* = 14 449), %^a^	Eczema (*n* = 4907), %^b^	OR	95% CI
Maternal education				
Degree plus	18·0	39·5	1·52	1·31–1·76
Diploma	9·4	39·0	1·49	1·25–1·78
A levels	9·7	35·4	1·28	1·09–1·51
GCSE grade A–C	35·7	34·5	1·23	1·08–1·40
GCSE grade D–G	11·0	33·6	1·18	1·03–1·36
None	16·1	30·0	–	–
Sex				
Male	51·1	36·2	–	–
Female	48·9	33·5	0·89	0·82–0·96
Ethnic group				
White	86·7	35·5	–	–
Mixed	3·3	37·4	1·09	0·85–1·38
Indian	1·9	30·6	0·80	0·61–1·07
Pakistani and Bangladeshi	4·1	21·6	0·50	0·42–0·60
Black or black British	2·8	37·1	1·07	0·82–1·41
Other	1·3	31·3	0·83	0·58–1·18
Maternal age at MCS child's birth (years)				
14–19	7·6	31·6	0·84	0·72–0·99
20–24	16·7	34·8	0·98	0·86–1·11
25–29	28·2	35·0	0·99	0·89–1·09
30–34	30·4	35·4	1·00	0·89–1·12
35–39	15	35·4	–	–
≥ 40	2·1	37·4	1·09	0·84–1·42
Maternal atopy				
None	70·8	31·1	–	–
Asthma or eczema	23·1	41·9	1·59	1·46–1·74
Asthma and eczema	6·1	52·8	2·48	2·13–2·88
Body mass index before pregnancy				
Underweight	5·4	31·1	0·80	0·65–0·99
Normal range	66·0	36·0	–	–
Overweight	19·7	35·0	0·96	0·86–1·07
Obese	6·5	36·3	1·01	0·82–1·24
Morbidly obese	2·4	33·7	0·90	0·70–1·16
Smoking status				
Never	65·1	35·7	–	–
Smoked before pregnancy	14·7	37·0	1·06	0·93–1·20
Smoked during pregnancy	20·2	31·1	0·81	0·73–0·91
Cigarettes per day during pregnancy				
0	78·2	36·3	–	–
1–9	11·5	33·0	0·86	0·75–0·99
10–19	6·7	30·3	0·76	0·64–0·91
≥ 20	3·6	31·2	0·80	0·64–1·00
Alcohol during pregnancy				
No	66·8	34·7	–	–
Yes	33·2	36·7	1·09	1·01–1·19
Birthweight				
Normal	93·9	35·6	–	–
Low	6·1	31·6	0·84	0·70–1·01
Caesarean section				
No	79·4	34·4	–	–
Yes	20·6	36·8	0·84	0·70–1·01
Breastfeeding				
Never	30·2	32·2	–	–
≤ 1 week	12·4	32·4	1·01	0·90–1·14
1–6 weeks	13·2	37·6	1·27	1·11–1·44
6 weeks to 6 months	16·6	34·1	1·09	0·97–1·23
≥ 6 months	27·6	38·2	1·30	1·16–1·47
Cow's milk at 9 months				
No	45·8	33·7	–	–
Yes	54·2	35·9	1·10	1·02–1·19
Solids before 4 months				
No	64·7	33·9	–	–
Yes	35·3	36·8	1·13	1·04–1·23
Antibiotics under 1 year				
No	50·1	32·2	–	–
Yes	39·3	38·9	1·34	1·22–1·48
Don't know	10·6	36·0		
ETS exposure in same room				
No	87·0	35·4	–	–
Yes	13·0	31·7	0·85	0·75–0·96
Exposure to grime				
No	78·4	34·5	–	–
Yes	21·6	36·7	1·10	1·00–1·21
Children in household				
1	42·1	36·5	–	–
2–3	50·9	34·2	0·90	0·83–0·98
≥ 4	7·0	30·8	0·77	0·65–0·92
Childcare				
None	68·2	33·9	–	–
Informal	18·3	35·5	1·07	0·96–1·20
Formal	13·4	38·7	1·23	1·08–1·39

CI, confidence interval; GCSE, General Certificate of Secondary Education; MCS, Millennium Cohort Study; ETS, environmental tobacco smoke. ^a^Percentage of total. ^b^Percentage with eczema in that group.

Figure 1 shows the unadjusted and adjusted covariate estimates (full data in Appendix S1; see Supporting Information). In the fully adjusted model, higher maternal qualifications, maternal atopy, breastfeeding pattern, cow's milk exposure at < 9 months, introduction of solids at < 4 months, antibiotic exposure in the first year of life and exposure to grime were associated with an increased OR for eczema. Female sex, Pakistani and Bangladeshi ethnicity, smoking during pregnancy, exposure to ETS and other siblings were associated with a reduced OR for eczema. There was no significant effect associated with preterm birth or furry pets, and these were not included in the final adjusted model.

### Association between maternal education and eczema, adjusted for other early‐life factors

**Figure 2:**
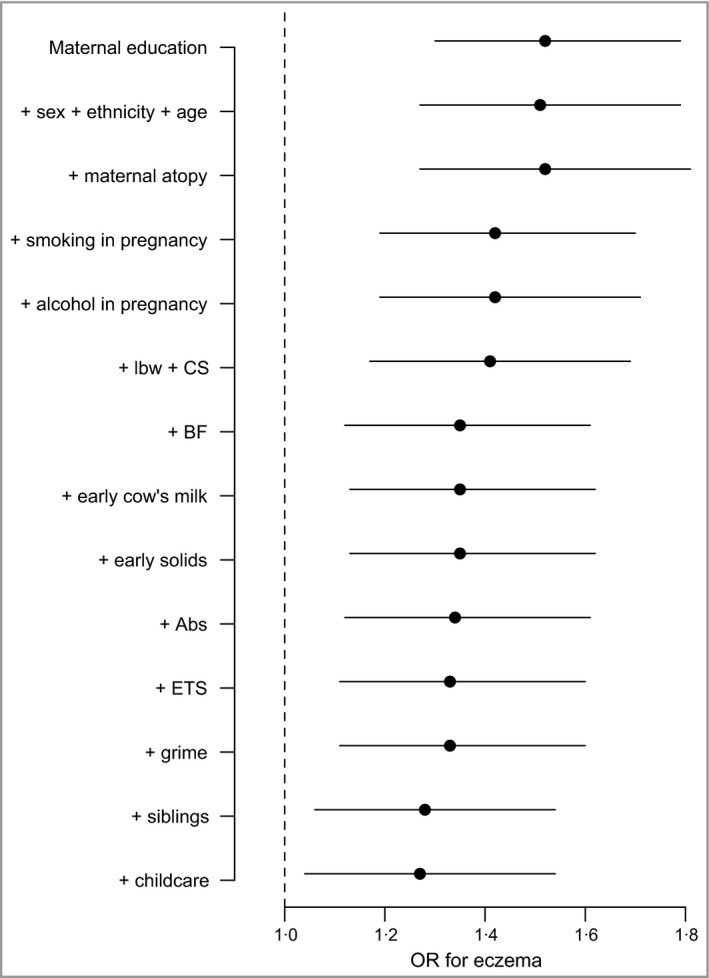
Odds ratios (ORs) for eczema comparing the highest maternal education group with the lowest in sequentially adjusted models. Sex + age + ethnicity has been added to the model as a block for the purposes of this figure. The plot shows partial attenuation of increased odds in children of more educated mothers with sequential adjustment for early‐life factors. lbw, low birthweight; CS, caesarean section; BF, breastfeeding; Abs, antibiotics; ETS, environmental tobacco smoke.

Figure 2 shows the extent to which the elevated OR of eczema in mothers with the highest educational qualifications [OR 1·52, 95% confidence interval (CI) 1·31–1·76], compared with mothers with no educational qualifications, attenuates when adjusting for other covariates. It shows the ORs for eczema after adjustment for covariates added sequentially using a life‐course approach (data tables showing all the model coefficients are provided in Appendix S1). The increased OR for eczema in children whose mothers have degree‐level qualifications remains after adjusting for sex, ethnicity, maternal age and atopy. In Figure 2 there are incremental changes in the OR evident after adjusting for smoking during pregnancy, breastfeeding pattern and exposure to other siblings. There are very small reductions associated with the variables of cow's milk, early solids, antibiotic use, ETS and grime. In the final full model the OR comparing the highest with the lowest qualifications remains significant (OR 1·26, 95% CI 1·03–1·50).

### Sensitivity analyses

Using income quintiles at 9 months (categorical variable), instead of level of maternal education, there was a similar pattern, with an increased OR for eczema as household income increased. However, all of this was attenuated when adjusting for the covariates in the full model (Appendix S1). The significant association between smoking during pregnancy and decreased odds of eczema remained evident when we added other measures of SECs to the final model, including income, occupational social class and family structure and income (OR 0·87, 95% CI 0·75–1·00). Repeating the analysis excluding the variables of maternal body mass index, alcohol during pregnancy, low birthweight and antibiotics raised the sample size to 13 747 (95%) and did not alter the conclusions. Repeating the analysis calculating relative risks rather than ORs did not alter the conclusions. Fitting the most parsimonious final model dropping nonsignificant variables did not alter the conclusions. Adding one‐way interactions between maternal education and other variables did not improve model fit.

## Discussion

Using a nationally representative sample of U.K. children we show that parental report of eczema at age 5 years is both common and socially patterned. Higher maternal qualifications, maternal atopy, breastfeeding at age > 6 months, introduction of solid foods at < 4 months, introduction of cow's milk before 9 months of age, antibiotic exposure in the first year of life and grime exposure are associated with an increased OR for eczema in the first 5 years of life. Female sex, Pakistani and Bangladeshi ethnicity, smoking during pregnancy, exposure to ETS and having other siblings were associated with a reduced OR for eczema. The increased odds of eczema with higher maternal education was attenuated when adjusting for early‐life factors.

Overall 35·1% of children had ‘ever had eczema’ by age 5 years. The high proportion of children who had ever had eczema by age 5 years reflects the cumulative nature of the phrasing of the question, and the fact that parental reporting is likely to be higher than doctor‐reported prevalence, which relies on health‐seeking behaviour and presence of visible eczema on visiting a doctor.

Our study corroborates a recent systematic review that collated and meta‐analysed observational studies that have assessed the association of socioeconomic position with risk of childhood eczema. Pooled estimates for the OR of eczema for the lowest compared with the highest socioeconomic position showed a reduced odds of eczema associated with lower socioeconomic position (e.g. the same direction of effect as in our study), and there was little change in the effect estimate in studies that adjusted for confounders or differences between groups (unadjusted OR 0·72, 95% CI 0·61–0·83; adjusted OR 0·79, 95% CI 0·73–0·85).^8^ Although some of the studies in the review adjusted for a range of variables, none attempted to explore factors that explain the positive social gradient.

Before we turn to the factors that partially attenuated the social patterning of eczema in our study, we will briefly review the main hypotheses raised in other studies included in the review by Uphoff *et al*.^8^ In one of the first studies to identify the unexpected social patterning of eczema, Williams *et al*. suggested that the reasons may include differences in the use of medications, carpets and heating influencing house dust mite populations, overuse of soaps, decreased exposure to ultraviolet radiation, increased contact with pets, and prenatal exposures correlated with higher social class, such as higher maternal age and maternal diet.^7^ While we did not have data on some of these variables, pets and maternal age did not alter the positive social gradient in the MCS. Artefactual explanations have also been suggested. If more highly educated mothers were more likely to have an atopic genetic background, the observed positive associations might be attributable in part to childhood genetic predisposition to allergic disorders.^20^ Furthermore, it might be that more highly educated parents are more likely to report eczema symptoms.

Breastfeeding (for 1–6 weeks and beyond 6 months) emerged as a significant risk factor for eczema in our study, and partially attenuates the positive educational gradient. We repeated the analysis treating breastfeeding as a continuous variable, and this suggested a linear relationship between weeks of breastfeeding and log‐odds of having eczema. We also explored the impact of exclusive vs. nonexclusive breastfeeding in a post hoc analysis. Exclusive breastfeeding increased the odds of eczema, but nonexclusive feeding did not (results not shown). Studies of the association between breastfeeding and atopic disease have produced conflicting results. A recent systematic review concluded that there was a lack of evidence for a protective effect of breastfeeding on eczema, and furthermore found a small increase in the risk of reported ‘eczema ever’ in association with ‘breastfeeding ever’ and breastfeeding < 6 months (pooled adjusted OR 1·11, 95% CI 1·00–1·22 and OR 1·10, 95% CI 1·02–1·20, respectively).^21^

Biological explanations for a relationship between breastfeeding and eczema risk have been suggested. For instance, prolonged breastfeeding may influence the development of tolerance to a permanent allergen‐specific intestinal immune response of the T helper 1 type at a critical developmental period.^22^ Furthermore, breast milk contains small amounts of immunogenic proteins transferred from the mother, and there are reports of eczema improving in breastfed infants when their mothers started an exclusion diet or stopped breastfeeding.^9^ Another possibility is reverse causation, whereby mothers choose to breastfeed for longer if they believe their child to be at high risk of eczema.

Smoking during pregnancy was associated with reduced odds of eczema in our study, and given the steep socioeconomic gradient in smoking during pregnancy, adjusting for this partially explained the higher prevalence of eczema in more advantaged children. Smoking in pregnancy has been identified as a risk factor for eczema in other observational studies, and potential biological mechanisms such as alterations in the T‐lymphocyte system or the respiratory tract mucosa have been raised.^23^, ^24^, ^25^ An analysis of linked routine data in Sweden by Hjern *et al*. found that children of mothers who smoked at least 15 cigarettes a day tended to have lower odds for having atopic eczema (OR 0·73).^26^ A large Danish study also showed a tendency towards a protective effect of smoking on hay fever and eczema.^23^ To explore this further we pooled the estimates from studies estimating the effect of maternal smoking during pregnancy on eczema risk in children. The overall estimate suggested a significant protective effect (Appendix S1), although several studies found no association and others found the opposite. This relationship may be due to residual confounding by other factors related to socioeconomic position, but the association remained evident even after adjustment for multiple measures of SECs.

Our study suggests that social patterning – in the early introduction of cow's milk and solid food, use of antibiotics in the first year of life, and exposure to passive tobacco smoke and pollution or ‘grime’ in the residential area – may make some contribution to the positive social gradient in eczema. Considering weaning patterns first, the times of exclusive breastfeeding and introduction of solids have long been recognized as important factors that may influence the development of allergy,^27^ and our study suggests that introduction of solid foods before the age of 4 months and the introduction of cow's milk before 9 months increase eczema risk. A systematic review has shown that use of antibiotics in the early years is associated with increased eczema risk, with a significant dose–response association, suggesting a 7% increase in the risk of eczema for each additional antibiotic course received.^6^ This may be explained by reverse causation, or confounding by indication, whereby antibiotics may be a consequence of infections in children with eczema. However, use of broad‐spectrum antibiotics may plausibly alter the gut microbiome, which plays an important role in the development of early immunity.^6^

Exposure to pollution or ‘grime’ in the residential area was socially patterned, and increased the risk of eczema. This variable is difficult to interpret, and may represent neighbourhood factors distant from the child rather than reflecting immediate household conditions. Our study suggests that exposure to siblings is protective for eczema. This goes some way to explaining the positive social gradient in eczema, because of the social patterning of larger families. This corroborates the original ‘hygiene hypothesis’, which was proposed based on the finding of a protective effect against hay fever from older siblings, the proposed mechanism being increased exposure to infections.^9^

Our study makes some additional contributions to the knowledge base around eczema risk factors. Female sex and Pakistani and Bangladeshi ethnicity were protective for eczema. The natural history of atopic eczema suggests a male predominance in childhood and female predominance from adolescence.^28^ There is little research exploring ethnic differences in the experience of atopic eczema during childhood within countries,^15^ and further research is needed to explain our findings regarding Pakistani and Bangladeshi ethnicity in the MCS. Our findings of no effect of furry pet exposure on eczema risk corroborate systematic review findings.^16^ Overall the review did not find any convincing evidence of an association between pet exposure and eczema risk. Some well‐conducted prospective studies in the review suggested that dog exposure might be protective against childhood eczema. To check this in the MCS we repeated our analysis using an indicator of dog exposure only, but this did not alter the conclusion (results not shown).

To our knowledge this study is the largest longitudinal study to explore the social patterning of eczema,^8^ and the first to identify early‐life exposures that can go some way to explaining the higher incidence in more advantaged populations. This study used data from a large, contemporary U.K. cohort, and the results are likely to be generalizable to other high‐income countries. A wide range of information is collected in the MCS, which allowed us to explore demographic, environmental and perinatal risk factors for eczema, including different measures of SECs. Eczema status was based on a question taken from the validated ISAAC questionnaire;^12^ however, like the other studies reported in this area, it based upon maternal report rather than a clinical diagnosis.

The major limitation of our paper is the self‐reported parental eczema outcome. Misclassification of the outcome is a concern, but we have used a validated eczema question, and previous studies support the use of parental report in aetiological cohort studies of eczema.^8^, ^29^ The cumulative nature of the question over a 5‐year period means that milder or short‐lived cases may have been forgotten. However, these are more likely to represent transient, more irritant forms of eczema, rather than atopic eczema, which is the target of this study. It is possible that mothers with higher educational qualifications are more likely to report eczema, for example due to greater exposure to health information. We believe this is unlikely to be the full explanation for the patterns seen in our study for three main reasons. Firstly, the same direction of association was observed across nine studies identified in the systematic review of Uphoff *et al*., in different settings and in studies that have used both parent‐ and doctor‐diagnosed outcome measures.^8^ Secondly, if mothers with higher qualifications were more likely to report eczema, one might expect the same to apply to other atopic conditions and parental self‐reported atopy. However, thirdly, a completely different social patterning of asthma, eczema and hay fever is evident in the MCS, and there is no social patterning evident in self‐reported parental atopy.

Missing data are a ubiquitous problem in cohort studies. Sampling and response weights were used in all analyses here to account for the sampling design and attrition. A complete case analysis was used, whereby individuals with incomplete data on covariates in the analysis were excluded from the analysis. This approach can be inefficient, which is a problem with smaller datasets, and may also introduce bias, when the individuals who are excluded are not a random sample from the target population. However, in this analysis the sample was large, and the internal associations, which were the targets of inference within the sample population, are likely to be valid, but we speculate that they may underestimate the effect sizes in the full U.K. population. However, repeating the analysis without some of the covariates where there were more missing data increased the sample to 95% of the cohort, and did not alter the main conclusions. We are mindful of the risk of spurious findings in observational studies and have been very careful to discuss our results from this study in the context of the global evidence base. We have not made corrections for multiple significance testing in our analysis, as these are rarely appropriate,^30^ particularly in the context of testing multiple substantively different hypotheses regarding different risk factors for eczema.

Although our analysis provides insights into the aetiology of eczema and its social patterning, it is challenging to identify tractable policy implications. Even if the association of smoking during pregnancy as a protective agent for childhood eczema is causal, there are no implications for tobacco control policies, as the established health risks of smoking will always be much greater than any potential benefits. The same applies to the promotion of breastfeeding; while current evidence does not support a protective role in breastfeeding for eczema, and indeed there is the suggestion of an opposite effect, the overall health benefits of breastfeeding are manifest. The link between increased risk of eczema and smaller families has long been established and is the basis of the hygiene hypothesis. Furthermore, some of the factors attenuating the increased risk of eczema in more educated families may not be causal, and may be related to a constellation of more proximal unidentified exposures that result in health impairment. The main implication from this study is to encourage more refined research into behavioural correlates of not smoking, smaller family size and prolonged breastfeeding to elucidate risk factors for eczema that might be amenable to public health interventions.

## Supporting information


**Appendix S1.** Supplementary data.
