# Supplementary material for: Do early‐life exposures explain why more advantaged children get eczema? Findings from the U.K. Millennium Cohort Study
Source: Br J Dermatol. 2016 Feb 23;174(3):569–78. doi: 10.1111/bjd.14310 (PMC4949701; doi:10.1111/bjd.14310)
Supplement: Supplementary file 1 — Appendix S1. Supplementary data. [file BJD-174-569-s001.docx]

**Web appendix: Do early life exposures explain why more advantaged children get eczema? Findings from the UK Millennium Cohort Study**

David C Taylor-Robinson^1,2^, Hywel Williams^3^, Anna Pearce^2^, Catherine Law^2^, Steve Hope^2^

**Web appendix: Odds ratio for eczema comparing highest income quintile to lowest in sequentially adjusted models.** Sex and age and ethnicity have been added to the model as a block for the purposes of this figure. The plot shows partial attenuation of increased odds in children of more educated mothers with sequentially adjustment for early life factors

**Web appendix: Effect of smoking during pregnancy on eczema in childhood**

To further explore the relationship between maternal smoking during pregnancy and childhood eczema we searched PubMed using the terms “maternal smoking AND pregnancy AND eczema”, and extracted and pooled effect estimates in the fixed effects meta-analysis below. Four other studies (Schafer 1997, Kipelainen 2002, Raherison 2007, Arshad 1992) reported no association, but did not provide estimates with measures of variance.

**Table:** **Fully adjusted associations (ORs) between covariates and eczema**

|  | Odds Ratio | Std. Err. | P>t | [95% Conf. | Interval] |
| --- | --- | --- | --- | --- | --- |
|  |  |  |  |  |  |
| **Maternal education** |  |  |  |  |  |
| Degree plus | 1.26 | 0.12 | 0.022 | 1.03 | 1.53 |
| Diploma | 1.22 | 0.13 | 0.061 | 0.99 | 1.50 |
| A levels | 1.04 | 0.10 | 0.66 | 0.86 | 1.27 |
| GCSE A-C | 1.06 | 0.08 | 0.437 | 0.91 | 1.24 |
| GCSE D-G | 1.03 | 0.09 | 0.764 | 0.86 | 1.22 |
|  |  |  |  |  |  |
| **Sex** |  |  |  |  |  |
| Female | 0.90 | 0.04 | 0.029 | 0.82 | 0.99 |
|  |  |  |  |  |  |
| **Ethnic group** |  |  |  |  |  |
| Mixed | 1.04 | 0.15 | 0.791 | 0.78 | 1.38 |
| Indian | 1.05 | 0.17 | 0.74 | 0.77 | 1.44 |
| Pakistani and Bangladeshi | 0.69 | 0.08 | 0.002 | 0.54 | 0.87 |
| Black or Black British | 1.35 | 0.18 | 0.03 | 1.03 | 1.76 |
| Other | 1.04 | 0.24 | 0.874 | 0.66 | 1.63 |
|  |  |  |  |  |  |
| **Maternal age at MCS child’s birth** |  |  |  |  |  |
| 14-19 | 0.94 | 0.11 | 0.613 | 0.75 | 1.19 |
| 20-24 | 1.04 | 0.08 | 0.583 | 0.90 | 1.21 |
| 25-29 | 0.97 | 0.06 | 0.573 | 0.86 | 1.09 |
| 35-39 | 1.00 | 0.07 | 0.94 | 0.88 | 1.14 |
| 40 and over | 1.16 | 0.17 | 0.306 | 0.87 | 1.56 |
|  |  |  |  |  |  |
| **Maternal atopy** |  |  |  |  |  |
| Asthma or eczema | 1.51 | 0.07 | 0 | 1.38 | 1.66 |
| Asthma and eczema | 2.49 | 0.22 | 0 | 2.09 | 2.97 |
|  |  |  |  |  |  |
| **BMI pre-pregnancy** |  |  |  |  |  |
| underweight | 0.85 | 0.10 | 0.147 | 0.68 | 1.06 |
| overweight | 0.97 | 0.06 | 0.639 | 0.87 | 1.09 |
| obese | 1.00 | 0.11 | 0.976 | 0.81 | 1.23 |
| morbidly obese | 0.90 | 0.12 | 0.442 | 0.69 | 1.18 |
|  |  |  |  |  |  |
| **Smoking status** |  |  |  |  |  |
| Smoked before pregnancy | 1.02 | 0.08 | 0.746 | 0.89 | 1.18 |
| Smoked during pregnancy | 0.86 | 0.05 | 0.014 | 0.76 | 0.97 |
|  |  |  |  |  |  |
| **Alcohol during pregnancy** |  |  |  |  |  |
| yes | 0.97 | 0.05 | 0.572 | 0.88 | 1.07 |
|  |  |  |  |  |  |
|  |  |  |  |  |  |
| **Low Birth Weight** | 0.85 | 0.08 | 0.101 | 0.71 | 1.03 |
| **Caesarean-section** | 1.05 | 0.06 | 0.402 | 0.94 | 1.18 |
|  |  |  |  |  |  |
| **Breast feeding** |  |  |  |  |  |
| One week or less | 0.98 | 0.07 | 0.756 | 0.86 | 1.12 |
| 1 - 6 weeks | 1.18 | 0.10 | 0.038 | 1.01 | 1.39 |
| 6 weeks - 6 months | 1.00 | 0.07 | 0.982 | 0.87 | 1.15 |
| 6 months or more | 1.17 | 0.09 | 0.038 | 1.01 | 1.36 |
|  |  |  |  |  |  |
| **Cows’ milk at 9 months** |  |  |  |  |  |
| Yes | 1.11 | 0.05 | 0.028 | 1.01 | 1.22 |
|  |  |  |  |  |  |
| **Solids before 4 months** |  |  |  |  |  |
| yes | 1.12 | 0.06 | 0.024 | 1.02 | 1.23 |
|  |  |  |  |  |  |
| **Antibiotics under 1 year** |  |  |  |  |  |
| Yes | 1.28 | 0.07 | 0 | 1.16 | 1.42 |
|  |  |  |  |  |  |
| **ETS exposure in same room** |  |  |  |  |  |
| Yes | 0.92 | 0.06 | 0.259 | 0.80 | 1.06 |
| **Exposure to grime** | 1.14 | 0.07 | 0.035 | 1.01 | 1.28 |
|  |  |  |  |  |  |
| **Children in household** |  |  |  |  |  |
| two or three.. | 0.90 | 0.04 | 0.04 | 0.82 | 1.00 |
| four or more.. | 0.84 | 0.09 | 0.102 | 0.68 | 1.04 |
|  |  |  |  |  |  |
| **Childcare** |  |  |  |  |  |
| informal | 1.04 | 0.07 | 0.558 | 0.92 | 1.18 |
| formal | 1.03 | 0.08 | 0.704 | 0.88 | 1.21 |

**MCS questions regarding atopy at sweep 5**

**
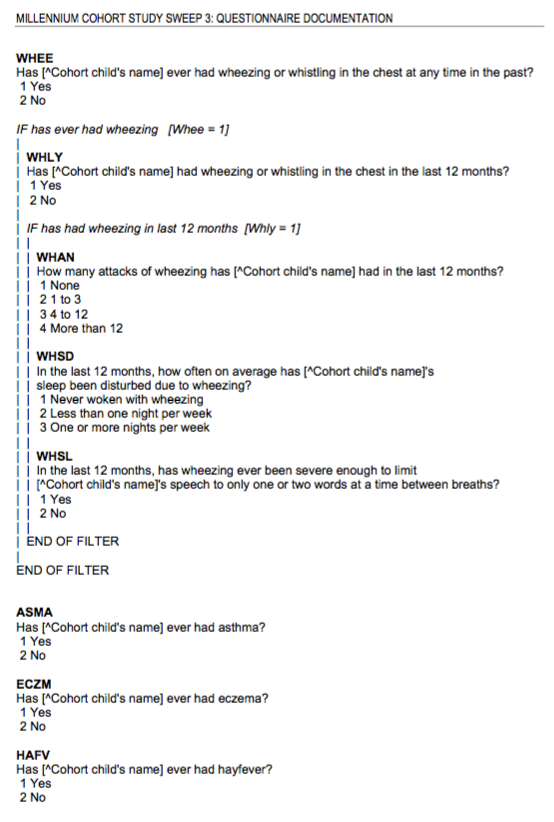
**
